# Sharka: The Past, The Present and The Future

**DOI:** 10.3390/v4112853

**Published:** 2012-11-07

**Authors:** Jiri Sochor, Petr Babula, Vojtech Adam, Boris Krska, Rene Kizek

**Affiliations:** 1 Department of Chemistry and Biochemistry, Faculty of Agronomy, Mendel University in Brno, Zemedelska 1, CZ-613 00 Brno, Czech Republic; sochor.jirik@seznam.cz (J.S.); petr_babula@email.cz (P.B.); vojtech.adam@mendelu.cz (V.A.); kizek@sci.muni.cz (R.K.); 2 Department of Natural Drugs, Faculty of Pharmacy, University of Veterinary and Pharmaceutical Sciences Brno, Palackeho 1-3, CZ-612 42, Czech Republic; 3 Central European Institute of Technology, Brno University of Technology, Technicka 3058/10, CZ-616 00 Brno, Czech Republic; 4 Department of Fruit Growing, Faculty of Horticulture, Mendel University in Brno, Valticka 337, CZ-691 44 Lednice, Czech Republic; Email: krska@zf.mendelu.cz

**Keywords:** viruses, plant, *Potyviridae*, *plum pox virus*, sharka, polymerase chain reaction, immune detection, ELISA, nanotechnology

## Abstract

Members the *Potyviridae *family belong to a group of plant viruses that are causing devastating plant diseases with a significant impact on agronomy and economics. *Plum pox virus* (PPV), as a causative agent of sharka disease, is widely discussed. The understanding of the molecular biology of potyviruses including PPV and the function of individual proteins as products of genome expression are quite necessary for the proposal the new antiviral strategies. This review brings to view the members of *Potyviridae *family with respect to *plum pox virus*. The genome of potyviruses is discussed with respect to protein products of its expression and their function. *Plum pox virus *distribution, genome organization, transmission and biochemical changes in infected plants are introduced. In addition, techniques used in PPV detection are accentuated and discussed, especially with respect to new modern techniques of nucleic acids isolation, based on the nanotechnological approach. Finally, perspectives on the future of possibilities for nanotechnology application in PPV determination/identification are outlined.

## 1. Introduction

**Figure 1:**
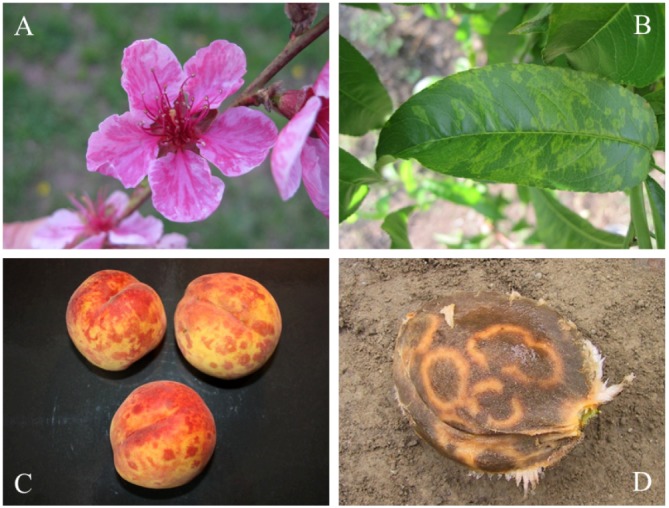
(**A**) Peach blossoms showing typical speckling of Plum Pox infected trees. Note the darker pink stripes on petals. (**B**) Chlorotic rings and blotches develop in peach leaves. (**C**) Yellow rings on a yellow-fleshed peach cultivar. (**D**) Ring patterns visible on the pit of an apricot.

Sharka or pox or plum pox disease is one of the most devastating viral diseases of stone fruits, with a significant impact on agronomy and economics. The disease is caused by *plum pox virus *(PPV), a member of *Potyvirus *genus in *Potyviridae *family. It is able to damage not only plums (especially *Prunus domestica *L.) [1,2], but also apricots (*Prunus armeniaca *L.), peaches (*Prunus persica *(L.) Batsch.) [3], nectarines (*Prunus persica *var. *nucipersica *(Borkh.) C.K. Schneid.*, *hybrids) [4], almonds (*Prunus dulcis *(Mill.) D. A. Webb*, *syn. *P. amygdalus *(L.) Batsch.*, Amygdalus communis *L., *Amygdalus dulcis *Mill.) [5,6,7], sweet cherries (*Prunus avium *(L.)) [8,9] and tart cherries (*Prunus cerasus *L.) [10,11,12]. PPV is able to infect also important ornamental and wild *Prunus *L species, including those used in traditional medicine: myrobalan (*P. cerasifera *Ehrh.) [13,14,15], American plum (*P. americana *Marsh.) [16,17], dwarf flowering almond (*P. glandulosa *Thunb.) [18,19], and blackthorn (*P. spinosa *L.) [17,20,21,22]. In addition, almost 60 species in eight families have been recognized as experimental host plants [23,24,25,26,27]. Nevertheless, *plum pox virus* is able to infect naturally occurring plants, which are not members of genus *Prunus*. Infected walnut trees (*Juglans regia *L.) were reported in Slovakia in 1996 by Baumgartnerova [28]. This information was based on the presence of diffuse spots on the leaves of infected plants. Despite the fact that PPV infection was detected by ELISA and the virus transmitted to the host plants, the presence of PPV-infected *Juglans regia *trees was not later confirmed, see Polak [29]. On the other hand, ELISA, ISEM and biological tests confirmed presence of PPV in *Euonymus europae *L. (*Celastraceae *R. Br.) and *Ligustrum vulgare *L. (*Oleaceae *Hffg. et Link) [29]. PPVis one of the most important members of family *Potyviridae* and the results of the infection are shown in Figure 1A-D*. *Family *Potyviridae* includes flexuous filamentous rod-shaped viruses of lengths of 650-950 nm and diameter of 12-15 nm, more exactly ssRNA non-encapsulated viruses with almost one-third plant viruses. The family includes seven genera as *Brambyvirus*, *Bymovirus*, *Ipomovirus*, *Macluravirus*, *Potyvirus*, *Rymovirus* and *Tritimovirus*. The most important members, their characteristics including vector(s), host plant(s) and distribution have been summarized in a plenty of extensive reviews, such as Gibbs and Ohshima [30], Gibbs *et al.* [31], Revers *et al.* [32], Riechmann *et al.* [33], and Ward and Shukla [34].

Potyviruses are transmitted predominantly by insects. Genera *Potyvirus *and *Macluravirus *are transmitted by aphids (*Aphis *Linnaeus*, Brachycaudus *van der Goot*, Brevicoryne *van der Goot, *Macrosiphum *Passerini*, Myzus *Passerini*, Phorodon *Passerini, and *Rhopalosiphum *Koch) [35,36,37,38]. In addition, eriophyid mites spread members of *Rymovirus *and *Tritimovirus *(*Abacarus *Keifer*, Aceria *Keifer) [39,40,41] and whiteflies (*Bemicia *Quaintance et Baker) spread members of *Ipomovirus * [42,43,44]. Only members of *Bymovirus *are transmitted by plasmodiophoroid fungus (*Polymyxa graminis* Ledingham, *Plasmodiophoridae*) [45,46,47,48]. It has been established that potyviruses are generally transmitted by non-circulative manner, which means that virus articles do not cross the vector cell membranes. However, not all potyviruses require specific associations with aphids. This exception is represented by *Bymovirus*, where species are restricted to *Poaceae *(*Graminae*) and are transmitted in soil by zoospores of *Polymyxa graminis* Ledingham. In this case, it is very difficult to study the epidemiology of diseases caused by *Bymovirus*, because they are not transmitted mechanically, purified preparations demonstrate only low infectivity, and the vector is an obligate parasite of roots that inhabits the individual cells of rhizodermis and cortex [49]. In addition, they frequently occur in a mixture with furoviruses. A new soilborne virus - *soybean leaf rugose mosaic virus* (SLRMV) - has been isolated and characterized by Kuroda *et al.* [50]. This virus is closely related to bymoviruses, however, further characterization is necessary. However, *Polymyxa graminis *is obligate parasite of monocot plants (*Glycine max *(L.) Merr. is dicot) and soybeans became diseased when grown in virus-infested soil [50]. With exception of *Bymovirus *with bipartite genome, where open reading frame is divided between two genomic RNAs, all potyviruses have monopartite genome with a genome-linked protein attached to the 5´ end (VPg) and a polyadenosine tail at the 3´ end of the genome. Their genome was characterised also as monocistronic for many years because of the presence of only one functional ORF. However, bioinformatic evidence and experimental verification of the evidence for overlapping coding sequences within the P3 cistron of potyviruses have been presented in the work of Chung *et al.* in 2008 [51].

**Figure 2:**
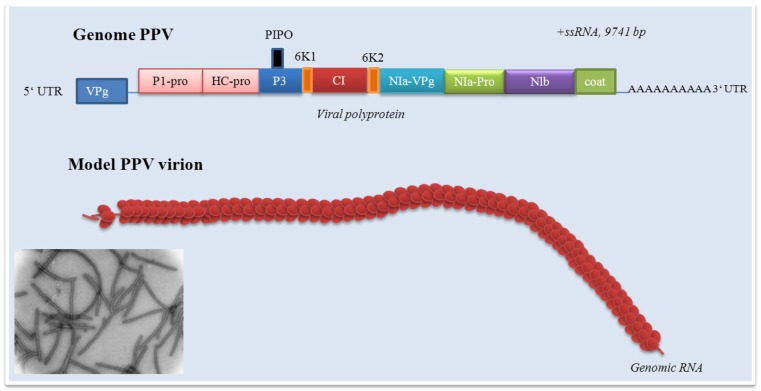
Potyvirus genome. CP: capsid protein; P1: P1 proteinase; HC-pro: helper component proteinase; P3: protein P3; PIPO: protein; CI: cytoplasmic inclusion protein; Nia-VPg: viral genome-linked protein; NIa-Pro: nuclear inclusion protein a; Nib: nuclear inclusion protein N (RNA-directed RNA polymerase).

In a Turnip mosaic virus, *pipo* (Pretty Interesting *Potyviridae* ORF) consists of 60 codons in the +2 reading frame relative to polyprotein, which places it within the P3 cistron. Disruption of *pipo *leads to the no changes in the polyprotein amino acid sequence; however, these mutants are not able to establish systemic infection. The ability of *pipo *mutants to establish systemic infection is still being discussed. Whereas Wen and Hajimorad in their mutational analysis of the putative pipo of *soybean mosaic virus* suggested that the disruption of PIPO protein impedes movement [52], the same authors together with Maroof later established that not PIPO but P3 is responsible for the changes in the virulence of the *soybean mosaic virus* [53]. A cap-independent translation is mediated by the 5´UTR [54]. The genome is expressed as a single polypeptide (polyprotein) precursor, which is proteolytically processed to mature virus proteins. However, due to fact that *pipo* that is predicted to encode a 7kDa protein was discovered to overlap with the P3 coding regions in all potyviruses. Immunodetection of the *pipo*-encoded protein in Turnip mosaic virus infected cells revealed a 25 kDa polypeptide, consistent with expression of *pipo* as a translational fusion with the N-terminus of P3 [51]. This fact must be carefully considered in the characterization of potyviruses genome organisation. The potyvirus genome consists of a positive-sense single messenger-polarity RNA molecule of the size of about 10 kb (generally 8.5 – 12 kb). The genome organization of potyviruses indicates nine cleavage sites and 11 mature proteins such as P1, HCpro, P3, P3N-PIPO, 6K1, CI, 6K2, NIa (respectively VPg and NIa-Pro), NIb and CP. In the light of above-mentioned fact, PIPO protein is expressed as a trans-frame protein consisting of the amino-terminal half of P3 fused to PIPO and a distinct C-terminus as a result of translational frameshift (P3N-PIPO). There are some exceptions in the potyviruses genome organisation [55]. *Ipomovirus *represents exception in the absence of HCpro and presence of two copies of P1 protein (P1a and P1b), which is shown in Figure 2. The nascent polyprotein precursor is cleaved by three viral proteinases (they are endopeptidases) as P1 (P1/HCpro cleavage site, serine proteinase with *cis-*cleavage activity), HCpro (HCpro/P3 cleavage site, HCpro cysteine proteinase with *cis-*cleavage activity), and NIa, respectively C-terminal proteinase domain NIa-Pro (NIa-Pro cysteine proteinase, all other cleavage sites) [56]. Potyviruses encode specific nonstructural protein called helper component (HC) that is obligatory for virus transmission by aphids. Its role probably consists in binding both to virions and to the cuticular lining of aphid mouthparts with retaining the virions within the maxillary food canal of the aphid stylets. This short-term reversible binding is most likely necessary and sufficient for successful virus transmission from one plant to other plants. However, above-mentioned facts are hypothetical and they have been not fully demonstrated. Functional proteins are generated after action of virus-encoded proteases. P1 proteinase is located at the very beginning of the viral genome of potyviruses. Due to the fact that the serine-like peptidase domain in the P1 coding region was identified in the C-terminal, highly conserved portion for all serine peptidases, associated with proteolytic activity and containing the Gly-Xaa-Ser-Gly motif, P1 is serine peptidase [57]. It varies in the length as well as in an amino acid sequence [58]. P1 poses RNA-binding activity and the possible influence in cell-to-cell viral spreading. Its role in defining the host plant is studied too [59]. Work of Shi *et al.* that showed interactions between P1 proteinase of *soybean mosaic virus *and Rieske Fe/S soybean protein are probably responsible for development of the symptoms of viral disease [60]. This N-terminal “hypervariable” protein is the most divergent protein among all potyviruses [61]. The *Tritimovirus* P1 functions as a suppressor of RNA silencing and an enhancer of disease symptoms were investigated by Young *et al.* [62]. However, P1 function must be further studied [63]. Potyviral helper component-proteinase (HCpro) has been recognized to be involved in the processes necessary for life, respectively infection cycle, especially in interactions with host proteins as well as other viral proteins. Thus, the presence of HC-pro is essential for all potyviruses [64]. These processes include not only interactions with host proteins, but also polyprotein processing and suppression of antiviral RNA silencing [65,66]. In addition, possible interactions between HCpro and host proteins must be carefully considered. This fact has been demonstrated in work of Shen *et al.,* who observed interactions between HCpro and full-length papaya calcireticulin, the multifunctional protein that regulates intracellular calcium(II) ions levels and protein folding in the endoplasmic reticulum [67]. In conclusion, HCpro has multiple functions. Despite the HCpro multiple function, the understanding of its function is still poor. The work of Guo *et al.* revealed the crystal function of cysteine protease domain of HCpro from *turnip mosaic virus* [68]. HCpro cleavages only dipeptide Gly-Gly at its C terminus. A cleaved C domain remains tightly bond at the active site cleft to prevent *trans* activity. Generally, the structure adopts a compact alpha/beta-fold. The catalytic cysteine and histidine residues constitute an active site and HCpro recognizes a consensus sequence YXGVV around the cleavage site between two glycine residues [68]. There are at least six cleavage sites in *cis*-/*trans*-arrangement in the viral polyprotein recognized by NIa proteinase (NIa-Pro). NIa-Pro represents C-terminal domain of NIa and is shown to be analogous to picornavirus 3C protease [69]. The N-terminal domain of NIa is designated as VPg and will be further discussed. The active – catalytic – sites of NIa-Pro are defined by a consensus heptapeptide sequence surrounding each cleavage site containing triad of His-Asp-Cys, which are conserved among the potyviruses [70]. The mutation of the catalytic residues His46, Asp81, and Cys151 resulted in complete loss of activity [71]. In addition, NIa proteinase possesses structural motifs shared with cellular serine proteases with the substitution of serine by a cysteine as the active site nucleophile. The specificity of enzyme and control its activity is discussed in some recently published papers [72,73,74], which are focused on the investigation of recombinant viruses and their host interactions. Mutation in the Lys(27) of NIa-Pro has established the role of this enzyme in the determination of host specificity. On the other hand, Lys(27) mutation did not affect the protease activity of NIa-Pro [75]. The NIa-Pro is a sequence-specific proteinase required for processing of viral polyprotein in the cytoplasm. This fact has been demonstrated in many potyviruses, such as *pepper vein banding virus* (PVBV), and is generally relevant for all known potyviruses [76]. Its accumulation in nuclei of infected cells manifested as a formation of “inclusion bodies” and has been demonstrated in some papers, for example see work of Anindya *et al.* [71] or Restrepo *et al.* [77]. This fact has been established by Knuhtsen, who observed tobacco etch virus-induced intranuclear inclusions [78]. However, this author is not the first to demonstrate the presence of nuclear inclusions in the nuclei of infected cells. In 1968, Shepard used electron microscopy for the characterization of nuclear (and cytoplasmic as well) inclusions in tobacco etch virus-infected *Nicotiana tabacum* L. cv. Havana 425 [79], and before him in 1941, Sheffied observed formation of nuclear inclusions in infected cells [80]. The NIa-Pro nuclear localization is still investigated. Accumulation of Nia-Pro in nuclei is observable mainly at the end of viral infection cycle. It seems that NIa-Pro executes DNA cleavage activity, respectively nonspecific double-stranded DNA degradation. This fact indicates the NIa-Pro role in viral infection cycle with subsequent degradation of the host DNA [71]. Potential use of NIa-Pro in biotechnologies is indicated in the paper by Fellers *et al.,* where three genes, each consisting of two NIa encoding regions (*tobacco vein mottling virus, tobacco etch virus *and *potato virus Y*) were introduced into *Nicotiana tabacum *L. cv. Burley 21. The authors indicate that “results of above-mentioned paper showed that different potyvirus NIa-Pro genes can be used for a protection against potyvirus infection and may protect plants against more than one potyvirus” [81]. Generally, transformation of plants with translatable sequences corresponding to the structural proteins, such as CP, or non-structural proteins such as the NIb or the P1 of potyviruses can make the plants highly resistant to infection with the corresponding virus [82,83,84]. Pathogen-derived resistance to viruses in transgenic plants is typically based on RNA silencing. This fundamental cytoplasmic antiviral defense system can be efficiently directed against viruses by producing homologous RNA from a transgene in plant cells. 

Plant potyviruses encode two membrane proteins 6K and P3. The role of P3 protein is only poorly understood. The role of P3 in viral infection cycle and as a symptom determinant is shown in the paper by Jenner *et al.* [85]. Development of symptoms of the disease may be closely connected with interactions between the P3 protein and proteins of host plant. The paper by Lin *et al.* demonstrates the possibility of interactions between the P3 protein encoded by *shallot yellow stripe virus *onion isolate and small subunit of RubisCo (ribulose-1,5-bisphosphate carboxylase oxygenase), enzyme, which is essentially involved in carbon fixation [86]. Point mutagenesis in the P3 *soybean mosaic virus* proved its role in virulence in Rsv4 genotype soybean. A single amino acid substitution (Q1033K) in the P3 led to the resistance towards Rsv4-mediated resistance [87]. The P3 protein role in virulence was confirmed by recently published papers [88,89,90,91]. The study of Cui *et al.* was focused on determination of the P3 protein role in *Nicotiana benthamiana *Domin (*Solanaceae*) leaf cells infected by *tobacco etch virus *(TEV) [92]. Authors carried out TEV subcellular localization. The P3 protein was localized in endoplasmic reticulum and formed punctate inclusions in association with Golgi apparatus. These structures originated directly from the endoplasmic reticulum exit side. Localization of P3 (and, in addition P3-6K intermediate) in the endoplasmic reticulum was previously reported by Eiamtanasate *et al.* [93]. Finally, the P3 punctate structure was found to traffic along the actin filaments and colocalize with the W-containing replication vesicles. Deletion analyses demonstrated the irreplaceable role of P3 domains in the retention of the P3 at the Golgi [92]. The 6K1 protein is generally quite insufficiently examined. In addition, data about its function is almost missing. The 6K1 protein is probably responsible for the cell-to-cell movement. This fact may be confirmed by the paper by Hong *et al.,* who observed the 6K1 protein at the cell periphery of infected leaves of *Pinellia ternata *(Thunb.) Ten. ex Breitenb. (*Araceae*) [94] and the paper by Saenz *et al.,* who determined 6K1 protein as the protein inducing viral replication vesicles [89]. However, the lack of a transmembrane domain suggests that it does not conform to recently recognized patterns of vital movement proteins [94]. The potyvirus cylindrical inclusion (CI) protein (or cylindrical inclusion helicase) represents a RNA helicase required for genome replication. The role of CI is discussed; however, this protein is necessary in facilitation cell-to-cell movement and replication. *Tobacco etch virus* (TEV) CI-mutants were replication-defective in protoplast inoculation assay, some mutants possessed cell-to-cell or long distance defects in the paper by Carrington *et al.* [95]. Ultrastuctural analyses revealed interactions between the CI protein and plasmodesmata and capsid protein-containing ribonucleoprotein complexes to make cell-to-cell and long-distance movement possible [96]. The presence of CI is connected with an apparent transient reduction in callose near the plasmodesmata [97]. Structural studies on *Nicotiana benthamiana* plants infected by *plum pox virus* revealed interactions between CI and the photosystem I PSI-K protein as the product of *psaK *gene. Coexpression of CI and PPV was shown to be responsible for a decrease of PSI-K accumulation as a result of *psaK* expression down-regulation. This effect is closely connected with PPV accumulation in *N. benthamiana *plants and confirms the role of CI in PPV infection [98]. In the recent papers, the role of CI in the breaking of resistance of plants against potyviruses is discussed [99]. The role of CI in the resistance against *lettuce mosaic virus *(LMV) is demonstrated in the paper by Tavert-Roudet *et al.* [100]. The C terminus of the CI together with VPg is directly involved in the overcoming *mo1*, the gene responsible for recessive resistance of plants to *lettuce mosaic virus*, resistance. *Mo1* gene encodes the eukaryotic translation initiation factor 4E (eIF 4E). This factor is a component of the eIF 4F complex and binds the cap structure of cellular mRNAs. Interactions between C-terminal part of the LMV CI and eIF 4E was proved [100]. However, further studies are necessary. Above-mentioned facts indicated the role of the viral protein linked to the genome (VPg), which represents the N-terminal domain of NIa. Its role, especially in resistant plants, is discussed in many published papers [101,102,103,104]. The VPg protein covalently links the 5´ end of the viral RNA via tyrosine residue. The *potato virus A* Vpg consists of 189 amino acids with a molecular mass of 21.9 kDa [105]. Its model suggests an elongated structure with a hydrophobic core composed of antiparallel beta-sheets surrounded by helices and a positively charged contact surface where most of the known activities are localized [106]. This protein also acts important roles in viral life cycle and enables systemic invasion. The VPg protein accumulation in companion cells (cells closely associated with sieve tube members, phloem) of infected leaves at the beginning of the systemic infection indicates the VPg as a “phloem” protein and its role in facilitating the virus unloading [107]. However, the questions focused on role of phloem, respectively sieve tube members, in plant viruses long-distance transport, must be further discussed [108]. A single amino acid substitution (His118Tyr) in VPg leads to the overcome of the strain-specific resistance to systemic infection and confirms the role of central part of VPg for systemic invasion of the potyviruses [109]. A Tyr63Ala mutation did not prevent replication completely but blocked spreading of the virus [106]. This protein was found to be phosphorylated as a part of the virus particle by the cellular kinase activity, which means that VPg is accessible to protein-protein interactions [110]. Interactions with HCPro were shown in the papers of Yambalo *et al.* and Roudet-Tavert *et al.* [111,112].

The potyvirus-interacting protein (PVIP), a plant-specific protein with some homologues has been identified in many plants. PVIP function is still discussed, however, as it probably serves as a control in chromatin remodelling and as an ancillary factor to support potyvirus movement in plants [113]. Co-immunoprecipitation and bimolecular fluorescence complementation (BiFC) assays revealed that P3N-PIPO interacts with host protein PCaP1, a cation-binding protein that attaches to the plasma membrane via myristoylation. PIPO domain of P3N-PIPO binds PCaP1; in this process myristoylation of PCaP1 is unnecessary for interaction with P3N-PIPO. It seems that PCaP1 links a complex of viral proteins and genomic RNA to the plasma membrane by binding P3N-PIPO, enabling localization to the plasmodesmata and cell-to-cell movement [55]. Phosphorylation itself probably plays important role in the VPg-mediated functions during the infection cycle of potyviruses [110]. Interactions between viral RNA polymerase and VPg support the VPg role in a viral RNA synthesis [114]. VPg displays still investigated roles in nuclei of host plants [115,116]. However, the nuclear VPg localization is not the only localization in infected cells. Using green fluorescent protein technologies, VPg was localized in endoplasmic reticulum with the probable role in viral RNA translation [117]. Mutants in the VPg domain demonstrate inhibition of nuclear transport debilitated viral genome amplification. Interactions between VPg and eIF 4E that indicate its role in the initiation of translation of the viral RNA has been demonstrated and represent a critical element for virus production [118,119,120,121,122]. VPg is probably an efficient modulator of eIF 4E biochemical function [123,124] and directs eIF 4E to promote viral RNA expression [125]. Interactions of VPg with next molecules, such as the phosphatidylserine of biomembranes [126]. However, the significance of these interactions must be further investigated. The NIb acts as a RNA-dependent RNA polymerase and is generally localized in the nuclei of infected plants [127]. Interactions between NIb and VPg have been demonstrated in some papers [128,129,130,131]. NIb role in uridylylation of VPg protein was investigated in *potato virus A *model and it seems that has important function in the regulation of RNA synthesis [131]. NIb introduction into plants (transgenic plants) may pose one of the directions in progress in the field of potyvirus-resistant plants [132,133].

The coat protein (CP) represents the multifunctional protein [134]. It is important for transmission of *Potyviridae *and, in addition, the degree of the identity between coat protein sequences can be used for the determination of relationship within the *Potyviridae * [135,136,137,138,139,140,141,142,143]. The role of CP and its binding with the helper component (HC) in aphid transmission is demonstrated [144,145,146]. CP-HC interactions are highly specific and require a Cys-Cys-Cys motif for specific interaction with CP Asp-Ala-Gly (DAG) motif [147]. Changes in DAG motif are the cause of systemic infection failure, but they do not affect replication and production of virions [148,149]. Further, N terminus of CP demonstrates next factor in the specificity for HC. This fact was confirmed by the work of Dombrovski *et al.,* who compared the differences between two potyviruses - *zucchini yellow mosaic virus* (ZYMV) and *turnip mosaic virus* (TuMV) with the same DAG domain [150]. Amino acid at the position 47 of the CP in the *pea seed-borne mosaic potyvirus* is highly important for ability of potyviruses to induce systemic infection [151]. However, due to the CP N-terminal region hypervariability, individual cases must be considered separately. CP is able to interact with NIb protein. However, the role of this interaction in the functioning of both proteins remains unknown [114]. Different plant viruses have been demonstrated to induce the expression of *HSP70*. This fact has also been established in some potyviruses, where up-regulation of *HSP70 *(*heat shock protein* gene)via a cytoplasmic unfolded protein response was established [152,153]. In addition, CP plays important role in these processes – increased amount of aggregated viral CP was observed under *tobacco mosaic virus* infection. Hafren *et al.* observed CP-mediated defect associated with replication under *HSP70* down-regulation. In addition, authors also propose the role of cochaperone CPIP in these processes [154]. The full-length *vanilla necrosis potyvirus* (VNV) coat protein (CP) gene was introduced into *Nicotiana benthamiana* plants via *Agrobacterium tumefaciens*-mediated transformation in the paper by Wang *et al.* [155]. Plants containing a full-length sense CP gene were highly resistant to virus infection. Similar papers demonstrating protective effect of CP gene expression in plants were demonstrated later [156,157]. However, the resistance is RNA-mediated. This circumstance is of high importance in biotechnology and engineering the virus-resistant plants [158,159,160,161,162]. However, virus resistance obtained by expressing the regions of a plant viruses genome in transgenic plants may be suppressed by the infection with another virus, which indicates the specificity in virus resistance [163]. On the other hand, the paper by Shand *et al.* points at the extensive cellular changes including disruption of the normal cell morphology, mitochondrial and chloroplast internal structures reorganization and formation of the atypical lipid deposits [164]. 

The 6K2 protein is as well as 6K1 protein only poorly understood. This transmembrane protein has no enzymatic function and enables anchoring the viral replication complex to membranes. It induces the formation of vesicles from endoplasmic reticulum membranes of the host leading to the formation of active replication compartments that contain all components required for virus replication. This fact has been confirmed especially by the papers of Restrepo-Hartwig *et al.,* who investigated a role of 6K2 protein in regulation of transport of the NIa via 6K2-NIa interactions to the nucleus [115,165,166]. Deletion or insertion of six histidine residues into various positions of membrane-bound 6K2 protein of *potato virus A *inhibited systemic infection independently in a host-specific manner [103,167]. Characterization and function of above-discussed proteins are summarized in Table 2.

## 2. Plum pox virus

The *plum pox virus* was firstly observed on plums in 1915 in Bulgaria and the disease was described in 1932 by Atanasoff [184]. Therefore, Eastern Europe was the localization of the first PPV epidemic. Later, PPV was reported on apricots (1933, by the same author who described the first PPV observation), on peaches (1961) and on sour cherries in the 1980´s [185]. Between 1932 and 1960, the disease caused by PPV moved from Bulgaria into Yugoslavia, Hungary, Romania, Albania, Czechoslovakia, Germany and Russia. Progression of disease in Western Europe was recorded after Word War II from Germany and Austria to The Netherlands, Switzerland, Greece, England and Turkey (1960s), then France, Italy, and Belgium (early 1970s), Spain and Portugal (early 1980s), Egypt, Syria and Cyprus (late 1980s). After 1990s, the disease was recorded in Chile, USA (Adams County, Pennsylvania, in 1999), Jordan, India and Canada. However, new reports about PPV occurrence (including individual PPV strains) still appear [186,187,188,189,190,191]. In Kazakhstan, PPV was firstly detected on plum and apricot trees in 2004 [192]. In 2005, *plum pox virus* has been found on apricot trees in China (Hunan Province of China, plants with typical yellow rings and diffused chlorotic spots) [193], in 2006, sharka disease in *Prunus* species was demonstrated in Argentina [194] and Pakistan [195]. One of the latest reports indicates detection of PPV in commercial Japanese apricot trees in Tokyo [196]. For the disease distribution and status, see Table 3.

PPV as a member of *Potyviridae, *which is a RNA virus with flexuous filamentous particles approximately 750 × 15 nm. It is widely distributed in Europe, North Africa, Asia and both Americas. As a natural host, PPV is restricted only to members of genus *Prunus *L. with above-mentioned exception. However, many experimental host plants have been identified. PPV is transmitted by several aphid species (see Table 1) [240]; however, it is also graft-transmissible to susceptible *Prunus *species and sap-transmissible to a wide range of herbaceous species. Symptoms of “sharka” highly depend on sensitivity of host plant and environmental conditions, especially on the weather conditions and age of the trees [241].

**Table 1:** Characterization of *Potyviridae *proteins including their function and subcellular localization.

Protein (size)	Function	Cellular localization	Reference(s)
P1 (32 – 64 K)	serine proteinase with *cis-*cleavage activity, P1/HCpro cleavage site. Responsible for **symptomatolology**. Usable for investigation of potyviruses phylogeny and relationship	Crystalline inclusions in cytoplasm and nuclei.	[56,61,63,168,169,170]
HCpro (56 – 58 K)	cysteine proteinase with *cis-*cleavage activity, HCpro/P3 cleavage site. **Aphid transmission, systemic movement, suppression of gene silencing, symptoms development.**	Cytoplasm, aggregates along the endoplasmic reticulum.	[82,171,172,173,174,175]
P3 (37 K)	plant pathogenicity – **virulence, viral infection cycle.**	Crystalline inclusions in cytoplasm and nuclei. Endoplasmic reticulum (punctuate inclusions in association with Golgi)	[86,91,92,169,176,177]
6K1 (6 K?)	cell-to-cell movement?, role in virulence?	The cell periphery.	[94,178,179]
CI (70 K)	RNA helicase required for genome replication. **Cell-to-cell movement. Long-distance movement. Development of symptoms. Breaking the host resistance**.	Cytoplasm, conical structures attached to plasmodesmata.	[95,97,98,99,180]
6K2	anchoring the viral replication complex to membranes. **Long-distance movement. Development of systemic infection**.	Crystalline inclusions in cytoplasm and nuclei.	[103,167,169]
VPg	covalently links the 5´ end of the viral RNA via tyrosine residue. **Viral cycle. Systemic infection. Overcoming the eIF4E-based recessive resistance**.	Endoplasmic reticulum, nucleus, nucleolus.	[100,102,104,114,117]
NIa-Pro	NIa-pro cysteine proteinase. Host specificity. Host DNA cleavage activity.	Nuclei of infected cells in the form of inclusion bodies.	[71,75]
NIb	RNA-dependent RNA polymerase. **Genome/viral replication.**	Nuclei of infected cells in the form of inclusion bodies. Cytoplasm.	[116,127,131]
CP	**Aphid transmission. Cell-to-cell and long-distance movement. Virion assembly.**	Plasmodesmatal connections between infected leaf mesophyll cells. Cytoplasm of infected cells.	[96,144,145,146,181,182,183]

**Table 2:** Sharka disease status in different countries. * - there are no data since 2003

Disease status	Country	Reference(s)
Widespread (high level)	Croatia, Greece, Bulgaria, Germany, Hungary, Poland, Romania, Russia, Serbia, Slovakia	[197,198,199,200,201,202,203,204,205,206,207,208,209,210]
Restricted distribution(heterogeneous levels)	Albania, Argentina, Austria, Canada, Cyprus, Czech Republic, Egypt, France, Italy, Iran *, *Kazakhstan, Luxembourg, Moldova, Norway, Pakistan, Portugal, Southern Russia, Slovenia, Spain, Syria, Turkey, Ukraine, United Kingdom, United States	[192,195,197,208,211,212,213,214,215,216,217,218,219,220,221,222,223,224,225,226,227,228,229]
Introduced, established	Azores, Bosnia-Herzegovina, Chile, Some states of former USSR including Central Asia, India, Jordan, Lithuania, The Netherlands, Switzerland, Tunisia	[197,208,218,230,231,232,233,234,235]
Introduced, eradicated	Belgium, Denmark	[197,208]
Not present	Estonia, Finland, Ireland, Israel, Lebanon, Malta, Morocco, Palestine, Sweden	[197,208,236,237]
Unknown status	China*, Libya	[208,238,239]

PPV usually affects both the leaves (leaf symptoms) and the fruit (fruit symptoms) of the plants. The intensity of fruit symptoms is usually significantly increased by the age of infected plants. The symptoms include chlorotic spots or rings, oak-leave patterns and vein clearing on leaves (plum), chlorotic pale-green rings and lines on leaves (apricot), small chlorotic blotches and distortion of the leaves (peach) or pale green patters and rings (cherry) [242,243]. Shallow rings and arabesque depressions, sometimes with brownish or reddish necrotic flesh (plums), light colored depressed rings (apricot), pale rings with diffuse bands on the epidermis (peach) or chlorotic and necrotic rings (cherry) are the main symptoms in the fruits [242]. In addition, fruits may drop prematurely [241]. Infection is usually symptomless in almond. On the other hand, *in vitro *explants lack the presence of symptoms typical for PPV infection; only typical interveinal chlorosis produced by PPV is visible [244]. This fact must be discussed in the light of the composition of cultivation medium, which represents complex matrix composed of macro- and microelements, as well as source of carbon (sugar(s)) and plant growth regulators - phytohormones. Just phytohormones play crucial role in the regulation of physiological processes as well as processes directing to the programmed cell death [245]. Most of the observable symptoms are probably dependent on a complex of virus-host interactions, where several or all viral genes could be involved in some way. On the other hand, the work of Clemente-Moreno *et al.* brings interesting findings and shows the possible protective role of ferrulic acid [244]. The work of Nagyova *et al.* revealed the role the 3´ proximal part of the *plum pox virus P1* gene in the virus-host interactions resulting in various pathotypes and demonstrated a different relative importance of particular PPV genes for symptom manifestation in different herbaceous host plant species [246]. There was an effort of several working groups to classify PPV in accordance with various characteristics, especially on the symptoms caused in experimental conditions of different plant hosts [27,247]. Based on different experimental plant hosts and symptoms, different strains have been described. Classification based on *Chenopodium foetidum* Lam. as an experimental plant host ranks PPV isolates to yellow, intermediate and necrotic strains [248]. Classification based on molecular-biological data initially brings the recognition of six strains of *plum pox virus*, see Table 4. Nevertheless, new isolates still occur and there is an effort for the establishment new PPV strains (for example “PPV-T, PPV-Penn, *etc*.”; however, these are still only isolates, for example PPV-Penn isolate(s) belongs to the PPV-D strain)[249,250]. On the other hand, PPV-EA isolate demonstrates 79–80 %, 80 %, 77 %, and 77 % homology with isolates of strains D/M, Rec, C, and W, respectively) and is classified as a strain [251,252]. The same situation is in the case of PPV-W that represents a new strain that occurs in Canada and Latvia [253].

**Table 3:** Classification of PPV strains, their first identification, characterization and distribution. * - isolate, ** - sour cherry isolate with unclear taxonomic relationship, in table as a strain. In addition, isolates are indicated in italics in the table.

Strain/Isolate*	First identification	Characterization	Distribution	References
PPV-M(Marcus)	Peach in northern Greece	Common strain in southern, eastern and central Europe. Spread rapidly by aphids, epidemic strain of PPV. Apricot, peach, plum.	Austria, Bosnia and Herzegovina, Bulgaria, Croatia, Czech Republic, France, Germany, Hungary, Iran, Italy, Jordan, Kosovo, Serbia, Slovakia, Slovenia, Spain, Romania, Turkey	[10,14,18,199,250,254,255,256,257,258,259,260,261,262,263,264,265,266,267,268,269,270,271]
PPV-D (Dideron)	Apricot in Southern France	The most common in western Europe, epidemic strain of PPV. Apricot, peach (only poorly), plum.	Bosnia and Herzegovina, Bulgaria, Canada, Chile, Croatia, Czech Republic, France, Hungary, Japan, Kosovo, Lithuania, Romania, Slovakia, Spain, Turkey, Ukraine	[18,19,196,218,254,258,265,268,271,272,273,274,275,276,277,278,279]
PPV-rec(recombinant)	Recombination between PPV-M and PPV-D	Widespread in several central and eastern Europe countries. Efficiently transmitted by aphids. Plums.	Bosnia and Herzegovina, Bulgaria, Croatia, Czech republic, Germany, Hungary, Italy, Kosovo, Serbia, Slovakia, Slovenia	[14,265,267,271,277,278,279,280,281]
PPV-EA(El Amar)	Apricot from Egypt	Only Egypt. Limited data about distribution. Apricot, peach, plum trees.	Egypt	[282,283,284,285,286]
PPV-C**(Cherry)	Cherry from Moldova	Eastern and central European countries, Italy (probably eradicated). Transmitted efficiently by aphids.Sweet (PPV-SwC) and sour cherry (PVP-SoC).	Hungary, Italy, Moldova, Romania	[287,288,289,290]
PPV-T* (Turkey)	*Plum from Ancara region, Turkey*	*Turkey, data still limited.*	*Turkey*	[250]
PPV-Penn* (Pennsylvania)	*Three different growers. Peach, nectarine, Plum.*	*USA, firstly in Adams County, Pensylvania in 1999.*	*USA*	[291,292]
PPV-W(Winona)	Plum from Canada	Canada, found in Latvia (The LV-141pl and LV-145bt isolates appear to be representatives of the "ancestral" PPV-W strain)	Canada, Latvia	[253,293]

## 3. Plum pox virus genome organization

PPV virions are long, flexuous and rod-shaped, approximately 750 nm (660-750 nm) in length and 15 nm (12.5 -20 nm) in width [294]. PPV as a member of *Potyviridae *has the structure typical for all potyviruses with exception of *Bymovirus. *The molecule of ssRNA is of positive polarity. The RNA of PPV has a VPg protein linked to its 5´end and a long poly(A) tail, which is heterogenous in its size at its 3´end [295]. In the case of an aphid non-transmissible PPV, excluding a 3´terminal poly(A) sequence the ssRNA is 9741 nucleotides in length. The 3´noncoding region is 220 nucleotides in length without the poly(A) tail [296]. PPV genome contains a long open reading frame starting at the first AUG codon, nucleotide 36, that is translated upon infection, starting at the second AUG codon as nucleotide 147, into a large polyprotein of 355.5 kDa [70,295,297]. There are still unanswered questions about initiation of potyviruses translation via recognition of the specific viral sequences [125,298]. Because potyviruses including PPV have relatively short 5´non-coding regions with a low content of cytosine and guanine, they avoid a stable secondary structures and lack nonfunctional intraleader opening reading frames [299,300]. Cap-independent internal initiation of translation has been proposed for four members of the genus *Potyvirus * [301,302]. Work of Simón-Buela *et al.* presents different *in vitro* and *in vivo* evidence of cap-independent leaky scanning as the mechanism of translation initiation of PPV genomic RNA [303]. Originated polyprotein precursor is co- and post- translationally cleaved by three virus-encoded proteinases into 11 mature proteins – P1, HCpro, P3, P3N-PIPO, 6K1, CI, 6K2, NIa (respectively VPg and NIa-Pro), NIb and CP. CP protein of about 36 kDa forms helically arranged “coat”. Similarly to other potyviruses, the PPV P1 proteinase represents together with P3/6K1, 6K2, NIa/VPg and N-terminal domain of CP highly variable protein [285,304,305]. This variability enables the determination of PPV isolates and their further characterization [285,304,305,306]. The untranslated region of PPV consists of 147 nucleotides, starting with four adenine residues. It seems that intact 5´end is not essential for PPV replication. PPV 5´ untranslated region that is essential for virus replication is confined to the first 35 residues. Deletion of a long sequence between nucleotide 39 and 145 did not affect the rate of infection and viral accumulation, but affected process of pathogenesis [307,308]. 

## 4. Plum pox virus transmission and cytological, histological and biochemical changes in infected plants

The PPV transmission is widely discussed in the two directions as aphid and non-aphid transmission. PPV is transmitted over short-distances in a non-persistent manner by *Aphis fabae *Scopoli (*Aphididae*), *Aphis gossypii *Glover(*Aphidae*), *Aphis spiraecola *Patch (*Aphididae*)*, Brachycaudus persicae *Passerini (*Aphididae*), *Myzus persicae *Sulzer (*Aphididae*) and next members of *Aphididae * [309,310,311,312,313]*.* Long-distance transmission is based on non-aphid transmission, this means on the spreading of infected plants and infected plant parts. The grafting may represent important risk in PPV spreading [24,314]. Milusheva *et al.* indicates the possibility of PPV transmission by infected seeds in a cultivar-dependent manner [315], however, some previously published papers disproves the possibility of PPV transmission by seeds [316,317]. The next fate of PPV and symptoms expression in infected plants is discussed. In stems of infected plants, PPV is localized in xylem and pith, which may indicate its possible spreading via xylem part of vascular tissue of infected plants [318]. This xylem transport does not suppose the transport for long distances, but for short distances (cell-to-cell) in the horizontal direction, which may be responsible for the localization of PPV in xylem and pith. This type of transport is provided by parenchyma cells. In addition, these cells are interconnected with other parenchyma cells; strict compartmentation is not possible. Later, this localization has been made more accurate. Hoffmann *et al.* proved the PPV presence in ray and axial parenchyma of vascular tissue of infected plants [319]. In petioles, PPV was demonstrated in epidermis and parenchyma cells of ground tissue, not in xylem [318]. These findings enable to express hypotheses about PPV spreading in plants [320,321]. On the other hand, there are significant differences between resistant and susceptible cultivars. Whereas the long-distance PPV spreading is highly limited in resistant cultivars, PPV susceptible cultivars allow long-distance PPV transport via xylem and the development of symptoms of sharka disease [320,322]. The differences between cell-to-cell (short-distance) and long-distance PPV transport between individual plant cultivars have been recorded, however, there are still unanswered questions [323]. *Plum pox virus* infection leads to the changes on different levels, *i.e*. cell and tissue as well as biochemical [1,324]. Whereas the changes in subcellular and cellular levels are connected with subcellular compartmentation of individual PPV proteins [320], changes in biochemistry of infected plants and resistant/susceptible plants may have practical impact in utilization of enzymes/proteins as markers of sharka disease. The paper by Hernandez *et al.* described the response of differently sensitive apricot (*Prunus armeniaca *L.) cultivars to *plum pox virus* infection [325]. This paper is interesting due to choice of cultivars, both resistant (Goldrich) and sensitive (Real Fino), and comparison of responses at antioxidant enzymes levels [325]. The most significant changes were observed in the case of superoxide dismutase(s) (SODs) and ascorbate peroxidase (APX) enzymes. Compared to sensitive cultivar, the significant decrease of APX activity in resistant cultivar was determined. Modifications in peroxidase activity were demonstrated also in *Nicotiana clevelandii *Gray PPV-infected plants, where the role of gaseous phytohormon ethylene in PPV-induced senescence has been partially revealed [326], and in *Chenopodium foetidum *Lam. [327]. On the other hand, levels of SODs, glutathione reductase (GR), dihydroascorbate reductase (DHAR) and monodehydroascorbate reductase (MDAR) of resistant cultivar were significantly increased compared to sensitive cultivar. Levels of catalase (CAT) remained unaltered. These results indicate the role of hydrogen peroxide, which is generated by the SOD activity, in response to PPV on the biochemical level. The experimental work continues in the second article of Hernandez *et al.,* in this case focused on the effect of PPV on photosynthesis and again on antioxidant enzymes [328]. These results were confirmed in pea plants (*Pisum sativum* L.), where PPV infection led to the accumulation of reactive oxygen species in chloroplasts under affecting of photosynthetic processes [329]. This fact is in agreement with papers focused on interactions between potyviral proteins and proteins of photosynthetic apparatus [98].The above-mentioned facts may be a reaction on its damage. In the third study, the same authors investigated response to long-term *plum pox virus* infection in peach plants (*Prunus persica* (L.) Batch cv. GF305 with the great susceptibility to PPV) with the focus on oxidative stress [328]. In this study, an oxidative stress and antioxidant mechanisms imbalance in accordance with the progress of PPV infection was determined. This fact has been confirmed by the following study of the authors [330]. Diaz-Vivancos *et al.* described an oxidative stress as a result of PPV infection in the apoplastic space of only susceptible apricot plants [331,332]. Whereas all above-mentioned papers were focused on oxidative stress caused by PPV infection, Escalettes *et al.* determined differential gene expression ins PPV-infected apricot cultivar (cv. Goldrich) [333]. The CH4 and CH22 fragments coding for a putative myosin and kinesin, were over-expressed in cv. Goldrich. Both myosin and kinesin are closely associated with cytokinesis, where they serve as motor proteins in the organization of phragmoplast microtubules. In the PPV-infected cv. Goldrich, the transketolase analogue CH15 was over-expressed, while it presented a very similarly modified expression in the susceptible genotype. Expression of the ankyrin-like CH27 was obviously enhanced in PPV-inoculated partially resistant apricot and in the susceptible Prunus genotype. The clear repression of CH29 transcript encoding a putative class III chitinase in the partially resistant genotype suggests a virus-mediated repression of this gene in the Goldrich cultivar [333]. *Arabidopsis thaliana* (L.) Heynh. was used as a model plant for investigation of gene expression alteration due PPV infection in the following study [334]. Genes associated with soluble sugar, starch and amino acid, intracellular membrane/membrane-bound organelles, chloroplast, and protein fate were up-regulated, while genes related to development/storage proteins, protein synthesis and translation, and cell wall-associated components were down-regulated. These gene expression changes were closely associated with PPV infection and symptom development [334]. The paper by Wang *et al.* revealed that genes involved in defense, cellular transport, development, protein synthesis, proteins with binding function in the PPV-infected peach leaf tissue are more active than those in PPV-free leaves [335]. The changes of expression of genes (increase of the following gene transcripts: including beta-1,3-glucanase, cytochrome-P450-like protein, cytochrome P450 monooxygenase, heat-shock protein 70, thioredoxin, alcohol dehydrogenase, catalase, cysteine protease inhibitor, translation factor EF-1 alpha, and pathogenesis-related protein (PRI)) in *Pisum sativum *L. PPV-infected plants support the ability of PPV to induce common stress responses in susceptible plants [335]. The recent studies focused on the changes in growth characteristics and yield of infected plants [336,337]. Similar results were demonstrated by Garcia-Ibarra *et al.,* who investigated changes in protective mechanisms including antioxidant enzymes in peach infected by *apple chlorotic leaf spot virus* (ACLSV, *Flexiviridae*). The results show increases in the APX, dehydroascorbate reductase (DHAR), superoxide dismutase (SOD) and glutathione S-transferase (GST) activities, whereas POX suffered a decrease of about 34 % [338]. Induction of oxidative stress by plant viruses is demonstrated in some works, such as Amari *et al.* [339], Clarke *et al.* [340], Farkas *et al.* [341], Fodor *et al.* [342], and Kiraly *et al.* [343]. In the lights of these findings, changes in markers of oxidative stress may be useful in the detection of PPV infection. However, further characterization of infected plants is necessary, because changes in antioxidant mechanisms under plant virus infection are indistinctive. 

## 5. Detection of PPV

The detection of PPV undergoes rapid development, from the usage of very simple methods based on host plants to molecular-biological methods and techniques. However, significant limitations for methods used in routine testing of plants for PPV must be carefully considered. These limitations are based especially on irregular distribution and translocation of PPV in plants in accordance with growth characteristics. Biological techniques are based on the infection and especially mechanical inoculation of susceptible plants - hosts, including both herbaceous and woody plants, from more than nine plant families (*Amaranthaceae *incl. *Chenopodiaceae*, *Cannabidaceae*, *Caryophyllaceae*, *Compositae* (*Asteraceae*), *Brassicaceae* (*Cruciferae*), *Fabaceae* (*Leguminosae*), *Ranunculaceae*, *Rosaceae* and *Solanaceae* [27,344,345]. Diagnostically important plant host species and symptoms are summarized in Table 4.

**Table 4:** Susceptible *Plum pox virus* (PPV) plant host species used in PPV detection and determination.

Plant host species	Symptoms	References
*Chenopodium foetidum *Lam.(*Amaranthaceae*)	yellow spots, some with necrotic centres or necrotic spots; not systemic	[327,346,347,348]
*Nicandra physalodes *(L.) P. Gaertn. (*Solanaceae*)	black-brown necrotic local lesions	[344,345]
*Prunus domestica *L. cv*. *Italian Pruneandcv. Pozegaca (*Rosaceae*)	chlorotic rings, bands and spots	[242,349,350]
*Prunus japonica *Thunb. (*Rosaceae*)	diffused chlorotic spots	[18,351]
*Prunus maritima *Marsh.(*Rosaceae*)	chlorotic spots, vein necrosis	[351]
*Prunus persica *L. (Batsch)cv. GF305	vein necrosis, malformation of leaves	[352,353]
*Prunus sibirica *L. (*Rosaceae*)	green spots and faint line pattern	[351,354]
*Prunus tomentosa *Thunb. (*Rosaceae*)	epinasty and malformation of young leaves, chlorotic spots, necrotic spots of older leaves	[23,355,356]
*Sorbus domestica *L. (*Rosaceae*)	yellow spots, leaf chlorosis	[208,357]

The first, but highly important step in progress of PPV diagnostics was based on the serological assay, respectively on the enzyme-linked immunosorbent assay (ELISA), which takes advantages from antibodies and their subsequent detection. One of the first record about the use of antibodies was published in 1980 [358]. ELISA method underwent progress with some modifications and now, it is one of the most popular and used techniques for PPV detection [359,360,361,362]. Whereas the usage of polyclonal antibodies, which recognize multiple epitopes on any one antigen, is controversial due to problems with specificity and consequently with sensitivity (serum contains a mixture of antibodies of different affinity), monoclonal antibodies, which detect only one epitope on the antigen, helped to overcome above-mentioned problems and are still widely used in PPV diagnostics [363,364,365,366,367]. Monoclonal antibodies are usually obtained after immunization of BALB/c mice with purified PPV isolates [367,368]. Despite the fact that commercial kits had been available and used before the introduction of 5B-IVIA monoclonal antibody, universal and specific detection of any PPV isolate was achieved by the use of 5B-IVIA monoclonal antibody [23,369]. Generally, this antibody enabled the production and use of commercially available kits for PPV detection, which enable specific identification of respective PPV strain. Antibodies are produced especially against non-structural PPV proteins as P3 [169], 6K2 [169], CP and CI [181,370,371], but also HCPro [181,372], NIb [169,181,373], and P1 [169]. However, non-structural proteins antibodies have not found the application in the practice and are used only for scientific purposes. As a secondary antibody, peroxidase-labelled, biotin-streptavidin system or fluorescence probe-labelled secondary antibodies are used [358]. Double Antibody Sandwich Indirect ELISA (DASI-ELISA) and Triple Antibody Sandwich ELISA (TAS-ELISA) have been recently recommended in PPV detection [13,23,266,269,360,374]. They are used especially for the determination of individual PPV strains [23,365,375,376]. Antibodies may be useful for the investigation of subcellular compartmentation of PPV mature proteins, or, in addition, may be used for subcellular localization of some members of *Potyviridae *family [181]. In this case, gold-labelled antibodies are used [169,377]. During the years, improved ELISA-based techniques have been proposed and developed. One of the most recent technique is impedimetric immunosensor, which is based on gold electrodes modified with 1,6-hexanedithiol, gold nanoparticles, anti-PPV IgG polyclonal antibody and bovine serum albumin. The proposed technique displays very good detection limit (10 pg/ml) and is able to differentiate the samples from healthy plants and the samples containing 0.01 % of infected plant extract [378]. The PCR-ELISA, respectively RT-PCR-ELISA, which is based on immunoenzymatic detection of PCR products, represents next possibility of ELISA/PCR modification with significant increase of sensitivity compared to both ELISA and PCR methods [374,379,380]. 

Hybridization techniques are based on an establishing a non-covalent, sequence-specific interaction between two or more complementary strands of nucleic acids into a single hybrid. The revolutionary were two works of Wetzel *et al.* published in 1991. Authors described molecular cloning and partially described the nucleotide sequence of the genomic RNA of PPV-EA. In addition, they compared this sequence to the corresponding sequence of previously sequenced PPV strains [381]. Finally, a sensitive, polyvalent assay based on the polymerase chain reaction (PCR) was developed for *plum pox potyvirus* (PPV) detection. This technique was adapted for a single tube, the chemical denaturation and reverse transcription of the viral RNA followed by the PCR reaction yielding a 243-base-pair product [382]. These two works started the large-scale application of PCR in the practice. Molecular hybridization techniques as well as different PCR-based assays have been developed to detect the PPV RNA presence in the sample [383,384,385,386]. PCR is widely used to amplify specific nucleic acid regions; thus, there is a necessity to have primers of known nucleotide sequence. In the past, different primers have been proposed and used, mainly on the HCPro [387], C-terminal part of NIb [265,388], C-terminal part of CP [249,265,389], N-terminal part of CP [390] or 3´-noncoding region [272]. The request to known nucleotide sequence is absolutely necessary also in hybridization techniques, which are popular due to relative simplicity. The paper by Pasquini *et al. *describes the strategy, development and use of 70-mer oligonucleotide probes specific for determination and genotyping (identification) the individual PPV isolates [391]. Despite the above-mentioned facts, the RNA isolation is the crucial step in these techniques. There are some difficulties connected with PPV RNA isolation from plant tissues, especially higher rate of polyphenolics and polysaccharides, which affect RNA isolation due to formation of complexes with both RNA and proteins. In addition, these compounds are able affect also activity of enzymes (including reverse transcriptase necessary for RNA transcription into cDNA form) used in PCR. One-Step RT-PCR as well as Two-Step RT-PCR is widely used in PPV RNA detection [265,392,393,394,395,396]. Products of PCR may be visualized by the electrophoretic separation of fragments in agarose gel with subsequent labelling by ethidium bromide or SYBR Green [397,398,399], on the nitrocellulose membrane [400], eventually by immunoenzymatic procedure [379]. There are some techniques, which are based on PCR technique and pose the improvement, especially significant enhancement, of sensitivity. Co-operational PCR (Co-PCR) has been described for sensitive detection of plant viruses and bacteria [401,402,403]. This technique, carried out in a single reaction, minimizes contamination risks and has a level of sensitivity similar to nested PCR and real-time PCR. In addition, it can be coupled with dot blot hybridization, making it possible to characterize the nucleotide sequence [401]. A highly sensitive assay, based on the polymerase chain reaction amplification of cDNA synthesized from the viral RNA of antibody-captured viral particles was developed by Wetzel *et al*. in 1992 [404]. The immunocapture step, by allowing the use of large sample volumes and by the viral particle pre-purification it achieves, dramatically increased the sensitivity of the assay. Recently, modifications of this technique are widely used. An immunocapture reverse transcription-polymerase chain reaction (IC-RT-PCR) based assay for the detection and identification of plant viruses represents technique using clarified plant extracts with degenerate primers, without necessity of isolation of total nucleic acids. This technique was used for detection and determination of *papaya ringspot virus * [405,406], *zantedeschia mild mosaic virus * [407], and *sugarcane streak mosaic virus * [408]. In addition, this technique was used in identification of PPV isolates [14,199,265,266,267,269]. A nucleic acid sequence-based amplification method coupled with flow-through hybridization (NASBA-FH) was developed for *plum pox virus* (PPV) detection with the detection limit 1000 times higher compared to conventional RT-PCR [409,410]. Despite the fact that ELISA followed by PCR techniques is the most frequently used technique in the PPV detection, loop-mediated isothermal amplification (LAMP) will probably become the most frequently applied approach in PPV detection. The great advantage of LAMP is its enormous rate of amplification paired with a very high specificity and low artefact susceptibility, which means great specificity [411]. Spot real-time RT-PCR is a method for detection of *plum pox virus* using conventional ELISA plant crude extracts immobilized on paper. This method has been developed to overcome the need of RNA isolation [412]. 

The field of nanotechnology is focused on the study and control of phenomena and materials and length scale below 100 nm, it means 1-100 nm [413,414]. This definition is not the only one, which tries to define nanotechnology. One of the most important criterions consists in accentuation the special properties of nanomaterials due to their nanoscaled proportions, which open their unique properties and features [415]. Nanotechnology found its application especially in industry and medicine, especially in electronics, imaging and drug delivery system for targeted therapy [416,417,418,419,420,421,422,423,424,425,426]. On the other hand, their application in the field of plant biology and phytopathology is limited, especially for cell imaging and manipulation [427]. There are works describing nanotechnology application in agriculture in the crop production in improving the yield and product quality [428,429,430,431,432]. On the other hand, plant viruses are considered as used in the nanotechnology, especially due to highly organized protein capsids, which are useful as scaffolds for building nanomaterials [433,434,435]. *Tobacco mosaic virus* has found applications as a building block for nanoelectronics as a template for metal deposition, mineralization and the deposition of the silica, such as nanowires and conductive films, as well as in light-harvesting systems [436,437,438,439,440]. *Cowpea mosaic virus* with its icosahedral protein coat shape can be utilized in non-invasive imaging, biosensors, and in vaccines [434,439,441]. Plant virus nanoparticles may be used in medicine in *in vivo *targeting and tumor targeting [442,443]. Based on the nanotechnology applications in the field of analytical chemistry [444,445,446,447,448], they represent the principal improvement of serological and immunofluorescence techniques in plant virus isolation and determination. In the field of PPV isolation and identification, there is an important requirement for PPV RNA of the highest quality. Therefore, methods of nanotechnology take advantage of nanoparticles on the basis of surface modifications possibility, which can significantly improve the quality of isolated RNA. Magnetic nanoparticles have been used for isolation and purification of nucleic acids; the use of magnetic nanoparticles provides very rapid and simple isolation of nucleic acids [449,450,451,452,453,454,455,456]. In addition, magnetic nanoparticled surface may be variously modified (these modifications include for example the introduction of silanol, epoxide, diol and carboxyl groups respectively). In comparison with traditional methods, the solid-phase process based on the interaction of nucleic acids with chemically-modified surface of magnetic nanoparticles is characterized by convenience, speed, timesaving, and being amenable to automation [457,458]. Using automation together with simple and rapid PPV RNA isolation under high specificity may represent great progress, especially due to the possibility of analyzing many samples in a short time period [459,460,461]. Generally, in the field of PPV detection, there is only limited number of publications focused on the use of nanoparticles in different isolation/detection steps. A paper by Byzova *et al.* introduces the possibility of using monoclonal antibodies as gold nanoparticle conjugates (26 nm in diameter) [462]. Using these conjugates with optimal ratio, an express immunochromatographic assay of PPV with a detection limit of 3 ng/ml and duration of 10 min. was developed [462]. Colloidal gold nanoparticles (5 – 60 nm) as a carrier system conjugated with corresponding antibody are used in the paper by Safenkova *et al.* [463]. This paper demonstrates correlation between gold nanoparticles, respectively conjugate size and affinity. An increase of conjugate size leads to the increase in its affinity [463]. In conclusion, not only improvement of those methods used (ELISA, PCR) and the establishment of new detection techniques, but also the development of new, effective methods usable in PPV RNA isolation with subsequent routine detection, will bring new opportunities to routine PPV detection, and thereby provide the possibility of eradicating plum pox disease as an one of the most devastating viral diseases of stone fruits. 
